# Effects of Low Frequency Residual Hearing on Music Perception and Psychoacoustic Abilities in Pediatric Cochlear Implant Recipients

**DOI:** 10.3389/fnins.2019.00924

**Published:** 2019-09-03

**Authors:** Mustafa Yüksel, Margaret A. Meredith, Jay T. Rubinstein

**Affiliations:** ^1^Audiology and Speech Disorders Program, Institute of Health Sciences, Marmara University, Istanbul, Turkey; ^2^Childhood Communication Center, Seattle Children’s Hospital, Seattle, WA, United States; ^3^Virginia Merrill Bloedel Hearing Research Center, Department of Otolaryngology – Head and Neck Surgery, University of Washington, Seattle, WA, United States

**Keywords:** cochlear implant, residual hearing, hearing preservation, music perception, psychoacoustics

## Abstract

Studies have demonstrated the benefits of low frequency residual hearing in music perception and for psychoacoustic abilities of adult cochlear implant (CI) users, but less is known about these effects in the pediatric group. Understanding the contribution of combined electric and acoustic stimulation in this group can help to gain a better perspective on decisions regarding bilateral implantation. We evaluated the performance of six unilaterally implanted children between 9 and 13 years of age with contralateral residual hearing using the Clinical Assessment of Music Perception (CAMP), spectral ripple discrimination (SRD), and temporal modulation transfer function (TMTF) tests and compared findings with previous research. Our study sample performed similarly to normal hearing subjects in pitch direction discrimination (0.81 semitones) and performed well above typical CI users in melody recognition (43.37%). The performance difference was less in timbre recognition (48.61%), SRD (1.47 ripple/octave), and TMTF for four modulation frequencies. These findings suggest that the combination of low frequency acoustic hearing with the broader frequency range of electric hearing can help to increase clinical CI benefit in pediatric users and decisions regarding second-side implantation should consider these factors.

## Introduction

Profoundly deaf patients can receive cochlear implants (CIs) and the advantages of electrical stimulation in restoring hearing capacity and speech understanding are well known. However, music perception and speech perception in noise are still generally poor in CI recipients without residual acoustic hearing, due to spectrotemporal limitations of electrical stimulation (Gfeller et al., 2002b, 2007; Nimmons et al., 2008; Won et al., 2010, 2011; Drennan et al., 2015). Combining acoustic hearing with electric stimulation, if available, can be advantageous and CI users who have residual low frequency hearing in the non-implanted ear or in the implanted ear can perform better on such tasks (Gantz and Turner, 2004; Gantz et al., 2006; Golub et al., 2012; Roland et al., 2016). There are different ways to achieve combined acoustic and electric hearing. CI users can use the residual hearing in the implanted ear when residual hearing is preserved, so called Hybrid (Cochlear Ltd., Sydney) or EAS (MED-EL, Innsbruck, Austria) hearing, or benefit from the residual hearing in the contralateral non-implanted ear, so-called bimodal hearing, with or without a hearing aid. The aim of both methods is to combine the high frequency information of electrical stimuli with the low frequency information of acoustic hearing to provide better spectral and temporal information via the combination than a CI can provide alone.

The benefits of combined acoustic and electric hearing are especially significant in music perception (Gfeller et al., 2006; Golub et al., 2012; Driscoll et al., 2016; Kelsall et al., 2017; Cheng et al., 2018; Parkinson et al., 2019). Gfeller et al. (2006) conducted two different experiments. In the first, authors compared 4 Hybrid CI users implanted with shorter electrode arrays with 39 CI users implanted with standard length electrodes and 17 normal hearing adults using an open-set melody recognition test. In the second, authors compared 14 Hybrid CI users implanted with shorter electrode arrays with 174 standard CI users implanted with standard length electrodes and 21 normal hearing adults using a closed set test of musical instrument identification. Hybrid CI users performed significantly better than standard CI users on both tests and similar to normal hearing subjects in melody recognition with lyrics. Dorman et al. (2008) evaluated and compared the melody recognition abilities of 15 CI users with and without a hearing aid in the non-implanted ear. Acoustic only (70.6%) and EAS (71.2%) modes were similar but the electric only mode (52%) was significantly poorer than the other two conditions. More recently, Kelsall et al. (2017) demonstrated that Hybrid CI users (*N* = 50) implanted with shorter electrode arrays performed similarly to normal hearing subjects in a pitch perception task using complex tones and the average score was 1.1 semitones. Conventional CI users tested in related studies have scores around three semitones. Melody perception was 65.9% and almost three times better than in conventional CI users. Timbre recognition was also better, but the difference was limited with Hybrid users scoring 56.6% and conventional CI users scoring 42.5%. The authors concluded that Hybrid CI users maintain music perception abilities postoperatively better than typically observed with conventional CIs.

To determine whether the preserved acoustic cues contribute to superior performance, Parkinson et al. (2019) examined the music perception of normal hearing subjects with acoustic simulations of Hybrid implants and compared electric only, acoustic only, and electro-acoustic conditions. Results showed better performance with the electric-acoustic condition (67.9%) in melody recognition scores compared to the electric-only condition (39.1%), but there was no effect of stimulation condition on timbre recognition scores. Researchers also compared the findings with the Hybrid L24 US clinical trial (Roland et al., 2016) and the results showed a similar pattern. The authors attributed the better melody recognition performance to the availability of low frequency spectral cues in the acoustic domain. In general, low frequency residual hearing appears highly beneficial for pitch and melody perception but less so for timbre perception.

Speech and musical sounds contain multiple frequency and timing cues that vary in a complex manner. Both normal hearing and CI systems need a detailed representation of acoustic signals in spectral and temporal domains to fully perceive such complex signals, but spectral and temporal sensitivity is limited in current implants as a result of biological and technological constraints. Won et al. (2010) evaluated the spectral and temporal sensitivity and music perception abilities of 42 adult CI recipients. Spectral sensitivity was shown to correlate with the three subtests of the Clinical Assessment of Music Perception (CAMP) test (Kang et al., 2009), but there was no correlation between temporal sensitivity and music perception. A similar study was performed with pediatric CI recipients (8–16 years of age) and the authors suggested that the spectral sensitivity might be decisive in the children’s performance (Jung et al., 2012). Heng et al. (2011) and Kong et al. (2011) both evaluated the relationship between timbre perception and temporal sensitivity in CI recipients and both studies emphasized the importance of temporal sensitivity in the perception of timbre. Recently Choi et al. (2018) evaluated the spectrotemporal modulation sensitivity and music perception of normal hearing listeners, hearing aid users, and CI recipients, and there was a significant correlation between music perception abilities and spectrotemporal modulation sensitivity, but there was no correlation between music perception and spectral or temporal modulation sensitivities alone. Golub et al. (2012) compared Hybrid CI recipients with standard CI recipients and pitch perception and spectral ripple discrimination (SRD) performance were significantly better in the Hybrid group and there was no advantage of residual acoustic hearing for temporal sensitivity.

Since there is no US FDA approval for the Hybrid or EAS systems for individuals younger than 18 years of age, reported outcomes in this age group with preserved residual hearing after implantation are limited. Benefit of combined acoustic and electric hearing on music emotion judgment (Giannantonio et al., 2015) and perception (Shirvani et al., 2016) and faster reaction time on music perception tests (Polonenko et al., 2017) were shown in bimodal CI users with a contralateral hearing aid. While these studies emphasize the benefit of combined electric and contralateral acoustic hearing, it is also possible to electro-acoustically stimulate the same ear and even the same single neuron (Tillein et al., 2015; Sato et al., 2017) with Hybrid and EAS systems. But there are only two studies directly evaluated the Hybrid or EAS system using subjects under 18 years of age. Driscoll et al. (2016) evaluated the music perception of five adolescents using Hybrid implants (13–18 years of age) with complex pitch ranking (PR-C), melodic error detection, and melody recognition. The performance of the Hybrid implant users on the three tests was significantly better than the traditional implant users and very similar to normal hearing peers. Recently Cheng et al. (2018) assessed the speech perception and melody contour identification of 35 children in unilateral (CI only) and bimodal (with hearing aid on the contralateral ear) conditions. Subjects performed significantly better in the bimodal condition on the melody contour identification and Mandarin tone recognition tests and results suggested that combined electric and acoustic hearing can improve both music and tonal speech perception in CI users.

Given the apparent benefits of bimodal hearing, there is an ongoing debate over when bimodal hearing may have benefits over bilateral CIs (Cullington and Zeng, 2011; Looi and Radford, 2011; Bartov and Most, 2014; Giannantonio et al., 2015; Polonenko et al., 2017; Gifford and Dorman, 2018). Studies reported that bimodal benefit is highly dependent on the degree of hearing loss, amount of pre-operative acoustic experience, and CI benefit (Looi and Radford, 2011; Illg et al., 2014; Dorman et al., 2015) and also the testing material used to evaluate the benefit. Standard clinical measures of speech perception alone are probably not reliable to determine second-side CI candidacy (Gifford and Dorman, 2018). Therefore, this study was intended to assess spectral and temporal sensitivity and music perception in older children with progressive hearing loss who have residual hearing in the contralateral ear to determine if, as expected, their outcomes were adult-like. We also intended to evaluate the benefits of residual acoustic hearing on music perception and spectral and temporal sensitivity in this age group. It is hoped that such measures can improve candidacy selection for second-side cochlear implantation in children with residual hearing in the unimplanted ear.

## Materials and Methods

### Subjects

**FIGURE 1:**
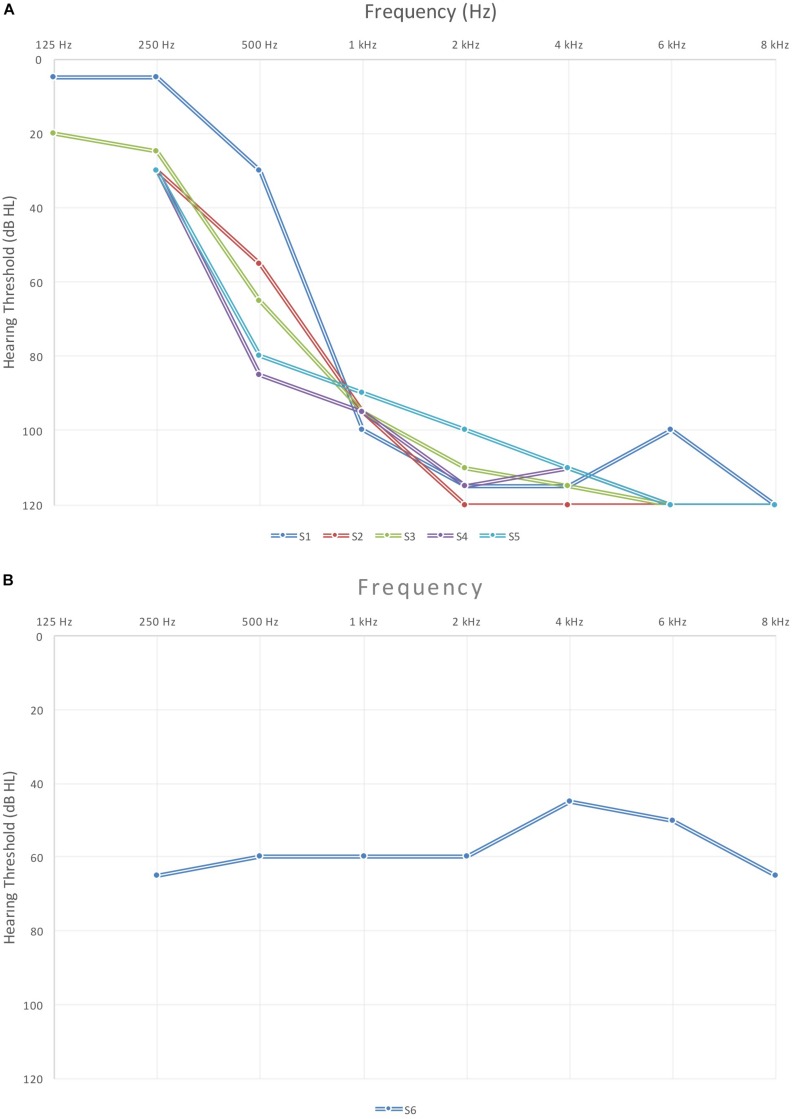
Audiometric configuration for unimplanted ears of study subjects: **(A)** five study subjects and **(B)** S6 with different configuration.

Six unilaterally implanted children between 9 and 13 years (*M* = 10.6 years) of age participated in this study. Subjects spoke English as their native language and had limited formal music training. Table 1 describes the demographics and etiology of hearing loss if known. All subjects passed newborn hearing screening bilaterally, have normal cochlear anatomy and early speech, and language development was normal by parent report. All subjects except S6 were deafened progressively while S6 had stable hearing thresholds through all of childhood. Mean age at diagnosis was 3.9 years and all subjects started to use hearing aids not later than 6 months after the diagnosis (mean age 4.1 years). Mean age at implantation was 5.2 years and all of the participants were using their implants for 4–8 years at the time of the study. All subjects received Cochlear Ltd. Devices using the Advanced Combination Encoder strategy at 900 Hz stimulation rate per channel. During the testing only one subject (S6) was wearing her hearing aid since she was the only one using a hearing aid in the unimplanted ear regularly. The other five subjects were not using an aid in the contralateral ear by the time of the study though some had earlier in their lives. Audiograms for the contralateral ear are shown for five subjects (Figure 1A). S6 has a significantly different audiometric configuration from the rest of the group, therefore we presented her audiogram separately (Figure 1B). The protocol was approved by the Seattle Children’s Hospital and University of Washington Institutional Review Boards and all subjects and their parents gave written informed consent.

**TABLE 1:** Demographics, etiology of hearing loss, and speech perception scores.

**Participant demographics**											

**Demographics**						**Cochlear implant**			**Speech perception scores**		
**No.**	**Age**	**Age at diagnosis (months)**	**Age at hearing aid (months)**	**Age at first CI (months)**	**Etiology**	**Device**	**Processor**	**Surgical approach**	**CNC (words) (%)**	**AzBio quiet (%)**	**AzBio + 5 SNR (%)**
1.	10	50	51	58	TMPRSS33 mutation	CI422	Nucleus 6	Round window	84	98	65
2.	12	25	28	46	TMPRSS33 mutation	CI512	Nucleus 6	Cochleostomy	80	95	42
3.	9	27	27	38	TMPRSS33 mutation	CI422	Nucleus 5	Round window	56	86	59
4.	11	75	76	84	Unknown	CI422	Nucleus 6	Round window	88	99	47
5.	11	75	76	84	Unknown	CI422	Nucleus 6	Round window	100	98	52
6.	13	51	53	60	Connexin26	CI422	Nucleus 5	Round window	80	90	66
Mean	11	50.5	51.83	61.67							

All tests were conducted in a sound-treated double walled room (IAC) with custom MATLAB programs and a sound field presentation level of 65 dB A. All stimuli were presented via a loudspeaker that was positioned at 0° azimuth and 0° elevation at a 1-m distance from the subjects.

### Clinical Assessment of Music Perception

Clinical Assessment of Music Perception is a test of music perception that was specifically developed for CI users and a previously described testing procedure was employed for pitch, timbre, and melody subtests (Nimmons et al., 2008; Kang et al., 2009). Each test was started with a training session that allowed the subjects to familiarize themselves with the task which allowing them to listen four different pitch pairs and 12 melodies and 8 instruments twice. During training sessions feedback was given, but in the actual testing no feedback was given to the subjects.

The complex pitch direction discrimination (PDD) test was a two alternative forced choice procedure in which the subject identifies the synthesized tone with a higher pitch. Two buttons (1 and 2) were presented on a computer screen and subjects were instructed to select the button corresponding to the tone with the higher pitch. Three base frequencies (262, 330, and 392 Hz) were used as a reference stimulus and in each tone pair the comparison stimulus interval was changed by the step size of one semitone. The initial pitch pair has an interval of 12 semitones and each correct response was followed by a smaller interval with incorrect responses followed by larger intervals. The discrimination threshold in semitones was estimated as the mean interval size for three base frequencies, each determined from the mean of the final six of eight reversals. A reversal at zero was automatically added by the test algorithm when the subjects answered correctly at a one semitone interval to create an accurate psychometric function.

Twelve well-known melody clips were played three times in random order in the melody recognition subtest. Rhythmic cues were removed from the melodies and each note of each melody has the same length and time signature with identical tempo. All melodies played in notes with a duration of 500 ms in an 8 note pattern at a tempo of 60 beats per minute. Each melody was prerecorded as five versions with different intensities per note (±4 dB) and each time a different version was played randomly to avoid intensity cues. After listening to the melody, subjects were asked to identify the melody by selecting the title of the song from the closed set of names. Measured outcome was a total percent correct score calculated after 36 melody presentations.

In timbre recognition, subjects were asked to identify a musical instrument from a closed set of names with the picture of the corresponding instrument. Eight instruments playing an identical five-note sequence were used and all instruments were recorded live with attempted identical phrasing. A total percent of instruments correctly identified was calculated after 24 presentations.

### Spectral Ripple Discrimination

The SRD test is an adaptive task and previously described stimuli and procedures were used (Won et al., 2007). The stimuli were generated by summing 200 pure tone frequency components with a duration of 500 ms and a rise/fall time of 150 ms. Each stimulus was either a standard (reference) or inverted ripple (ripple phase – reversed). The stimuli had a bandwidth of 100–5000 Hz and the ripple densities differed by ratios of 1.411. The test paradigm used a two up and one down adaptive forced-choice procedure and spectral ripple resolution thresholds were determined converging on 70.7% correct (Levitt, 1971). Thresholds were determined as the highest ripple density (in ripples per octave – rpo) at which listeners were able to discriminate an inverted signal from two standard stimuli, identical to the inverted one except that the positions of spectral peaks and valleys were reversed. Subjects were instructed to select the respective number of different/inverted signal from computer screen. Testing included three adaptive tracks. The mean threshold of a single track was determined by averaging the final 8 of 13 reversals. The final SRD thresholds were determined by averaging three adaptive tracks. A few examples were shown to subjects prior to the actual test until the examiner is confident the subject understood the task and as the actual testing began no feedback was given.

### Temporal Modulation Transfer Function

The temporal modulation transfer function (TMTF) test used in this study was adapted from Bacon and Viemeister (1985) and modified by Won et al. (2011). Stimuli consisted of two intervals, one with unmodulated and one with sinusoidally amplitude modulated (SAM) wide-band noise and subjects were instructed to choose the interval with the modulation. Total duration of the stimuli was 2 s and each interval was 1 s. Modulation frequencies of 10, 50, 100, and 300 Hz were used to keep the attention of the subjects since three tests were taking approximately 90 min and this also allowed analysis of the high pass characteristic of the TMTF. Modulated stimuli were created with the following equation:

$$y\,\left(t\right)=\left[f\,\left(t\right)\right]\,x\,\left[1+m_{i}\,\sin\left(2\,\pi \,f_{m}\,t\right)\right]$$

In this equation, *t* indicates time and *x* indicates multiplication, *f*(*t*) is the wideband Gaussian noise carrier, *m*_*i*_ is the modulation index (modulation depth), *f*_*m*_ is the modulation frequency, and *y*(*t*) is the resulting signal. To compensate the intensity increment for the SAM stimuli the modulated waveform was divided by a factor of 1 + ($m_{i}^{2}/2$). A two interval two alternative forced-choice test with two down one up adaptive procedure was used. Measured outcome was the modulation depth (*m*_*i*_) threshold (MDT) for each modulation frequency, converging on 70.7% (Levitt, 1971). The starting modulation depth was 100% and decreasing in steps of 4 dB for the first four reversals and 2 dB for the next 10 reversals. MDT for each modulation frequency was obtained from the average of the final 10 reversals. Reported values are in dB relative to 100% modulation depth.

## Results

Since our study sample was small, results from previous studies conducted with the same testing material in the same research center were used as a reference and values are shown in the respective figures.

### CAMP

**FIGURE 2:**
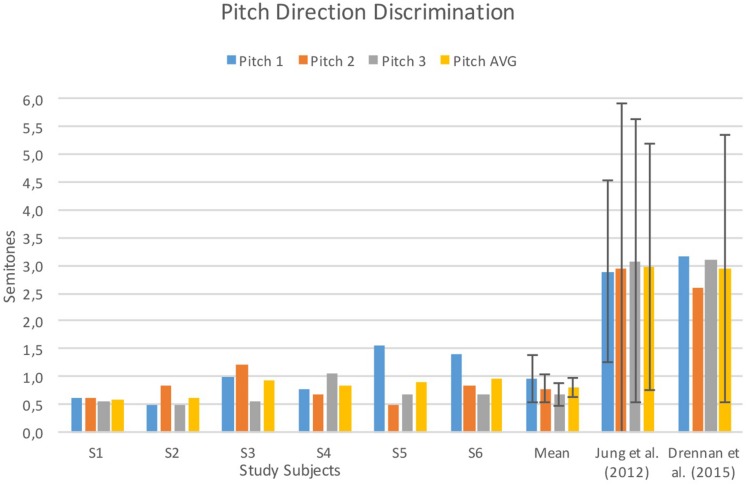
Individual and mean pitch direction discrimination scores for study subjects and mean scores from previous studies. Error bars indicate ±1 standard deviation. AVG, average. Data adopted from Jung et al. (2012) and Drennan et al. (2015).

**FIGURE 3:**
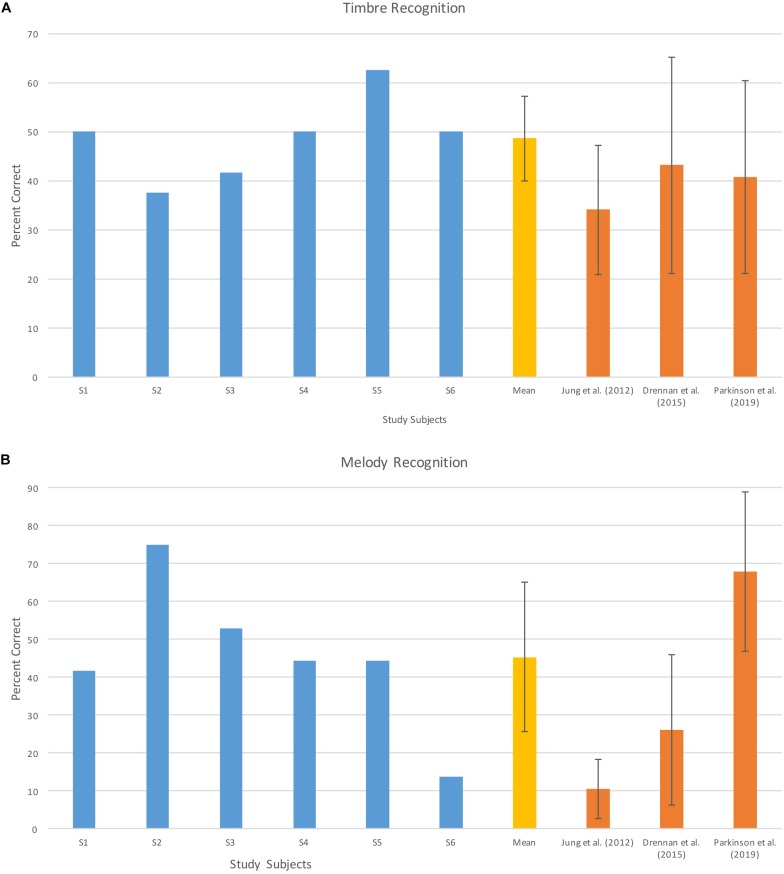
Individual and mean timbre **(A)** and melody **(B)** recognition scores for study subjects and mean scores from previous studies. Error bars indicate ±1 standard deviation. Data adopted from Jung et al. (2012), Drennan et al. (2015), and Parkinson et al. (2019).

Pitch direction discrimination, timbre recognition, and melody recognition scores of six study subjects and mean values from previous studies conducted with normal hearing subjects and standard length electrode CI users (Kang et al., 2009; Jung et al., 2012; Drennan et al., 2015) for reference are shown in Figures 2, 3.

Mean PDD thresholds for the study sample were 0.97 (±0.43) for 262 Hz; 0.78 (±0.25) for 330 Hz; 0.67 (±0.20) for 391 Hz; and 0.81 (±0.16) semitones on average. Jung et al. (2012) assessed 11 prelingually deafened pediatric CI users with standard length electrodes and no residual hearing and subjects scored 2.98 (±2.23) semitones on average and 145 postlingually deafened adult CI users in the Drennan et al. (2015) study scored 2.95 (±2.40) semitones on average (Figure 2). Mean PDD threshold of normal hearing subjects in Kang et al. (2009) study was 1.0 (±0.03).

The timbre recognition score of the subjects was 48.61% (±8.06) on average. Pediatric users of CIs without residual hearing in a previous study scored 34.09% (±13.15) on average (Jung et al., 2012). Adult CI recipients in the Drennan et al. (2015) study and adults with Hybrid devices in electro-acoustic mode in Parkinson et al. (2019) study scored 43.2 (±22%) and 40.7 (±19.7%), respectively (Figure 3A). Mean timbre recognition of normal hearing subjects in Kang et al. (2009) study was 94.2 (±4.0%).

On the melody recognition subtest the subjects scored 43.37% (±19.49) on average and with that score our study sample performed almost four times better than the children from a previous study without residual hearing (Jung et al., 2012). In reference studies adult CI users scored 26.20% (±19.90) on average (Drennan et al., 2015) and adult Hybrid users in the electric-acoustic condition in the Parkinson et al. (2019) study scored 67.90% (±21.10) on average (Figure 3B). Mean melody recognition of normal hearing subjects in Kang et al. (2009) study was 87.5% (±8.3%).

### Spectral Ripple Discrimination

**FIGURE 4:**
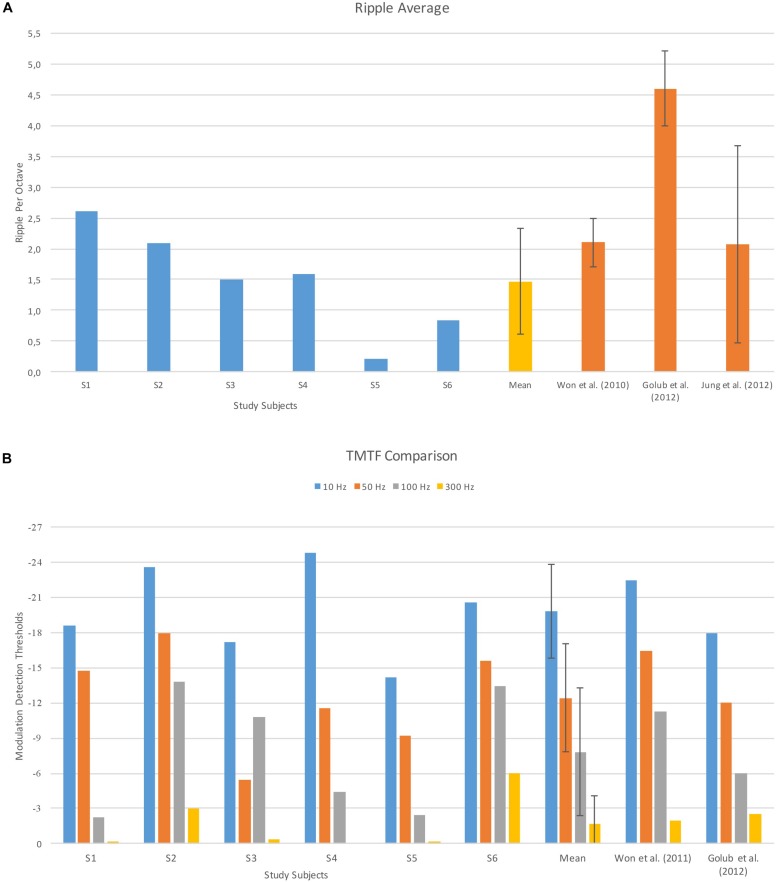
Spectral ripple discrimination **(A)** and TMTF **(B)** performance of study subjects and mean scores from previous studies. Error bars indicate ±1 standard deviation. Data adopted from Won et al. (2010), Golub et al. (2012), and Jung et al. (2012).

Mean spectral ripple threshold of study subjects was 1.47 (±0.85) rpo. Pediatric and adult CI recipients and adult Hybrid CI recipients in previous studies have mean spectral ripple thresholds at 2.08 (±1.6), 2.10 (0.40) and 4.60 (±0.60) rpo, respectively (Figure 4A).

### Temporal Modulation Transfer Function

Temporal modulation detection thresholds in four modulation frequency are shown in Figure 4B. Two previous reference studies were conducted with adult CI recipients (Won et al., 2011; Golub et al., 2012) but the values were quite similar. The low pass shape of the present study’s TMTFs is similar to TMTFs measured in previous studies.

## Discussion

This study demonstrates the benefit of low frequency residual hearing on music perception measurements in CI recipients. Such benefit was shown in previous studies with bimodal users (Bartov and Most, 2014; Cheng et al., 2018), Hybrid CI recipients (Driscoll et al., 2016), and acoustic simulations of the electric-acoustic condition (Parkinson et al., 2019).

The complex PDD performance of our study sample was very close to normal hearing subjects in previous studies (Gfeller et al., 2002a; Kang et al., 2009) with values ranging between 0.59 and 0.96 semitones. Adolescent Hybrid CI users in the Driscoll et al. (2016) study performed between 1 and 6 semitones but the PR-C Test was used in that study and the best score in PR-C is 1 semitone while the best score in PDD of CAMP is 0.50 semitones. Also the participants in our study had progressive hearing losses but there was no comparable information given in the Driscoll et al. (2016) study. Adult Hybrid CI users in the Kelsall et al. (2017) study performed the same pre (with hearing aids) and post operatively (mean = 1.1 semitones) in the PDD test and this observation is an important evidence for the benefit of low frequency hearing on pitch perception. This benefit is especially significant, since the pitch range evaluated in CAMP is a direct reflection of western musical instruments and melody intervals that mainly focused on octaves around middle C (262 Hz). In the other two measurements of music perception, the study sample performed better than CI recipients without any acoustic hearing, particularly for melody recognition. We believe that the superior performance in the melody recognition can be related to the PDD, since the melodies were sequential tones with changing pitches in an isochronous manner and as mentioned earlier, these pitches are well within the residual hearing range of subjects 1–5. Unlike the PDD test, the participants did not reach the normal range in the melody recognition test. This finding is consistent with Jung et al. (2012) study where child CI users with standard length electrode arrays performed similarly to adults in the PDD test but performed below chance level on the melody recognition test. It should be considered that perception of melodies requires more attention and memory-related performance compared to the PDD test and this requirement might be dominant for children. Performance was significantly worse than normal hearing in timbre recognition. Timbre of musical instruments is a perceptual concept related to salient peaks on the spectral envelope that can present itself as formants or harmonics (Burred et al., 2006) in the upper frequency range depending on the resonance characteristics of respective instruments (Toole and Olive, 1988). Therefore, the timbre recognition task is more related to broadband perception than pitch and melody recognition tasks and hence dependent on the well-known spectral limitations of CIs.

The superior performance seen in music perception was not evident in SRD and TMTF measurements. The SRD test stimulus has a frequency range of 100–5000 Hz and the perception of high frequency information above 1000 Hz is critical for ripple detection. Effects of age and maturation were studied previously and it has been suggested that SRD matures at 7 years of age in CI recipients (Horn et al., 2017). This is based not only on chronological age but also on CI age (DiNino and Arenberg, 2018) for congenitally deaf children. Our study sample consisted of children with progressive hearing loss who may develop normal central auditory function regardless of implantation age (Sharma and Dorman, 2006). Also, Jung et al. (2012) found no difference between adult and pediatric CI recipients in SRD testing. Therefore, we can speculate that the absence of difference can be expected due to the hearing loss above 1 kHz. In the same study by Jung et al. (2012) the difference between Schroeder-phase discrimination scores at 50 and 200 Hz of adult and pediatric CI groups was statistically significant and it is known that temporal auditory processing can be influenced by age (Brennan et al., 2018), become adult like at age of 11 years in normal hearing individuals (Buss et al., 2017) and development of temporal modulation sensitivity is delayed in pediatric CI users (Park et al., 2015). The precise maturation process in implanted children, and the effects of residual hearing and progressive hearing loss are still not known, but the effect of age and maturation must be considered when interpreting our results.

### Limitations of the Study

Our sample size was small due to the specific nature of the CI recipients in this study. This limitation precludes detailed statistical analysis. It is believed, however, that the findings present valuable information to assist decisions on when to perform second-side implantation in older children with residual hearing.

## Conclusion

Our study sample performed similarly to normal hearing listeners in the PDD task and also performed substantially better on melody recognition than CI recipients without residual hearing in previous studies. Timbre recognition, SRD, and TMTF performances were similar to previous studies of implant recipients. These findings must have taken into account when deciding whether to proceed with second-side implants in children with residual hearing. CI recipients who perform similarly to normal hearing individuals in any behavioral measure is an important and atypical outcome measurement post implantation, hence preserving this ability should be a priority for clinicians.

## Data Availability

The datasets generated for this study are available on request to the corresponding author.

## Ethics Statement

This study was carried out in accordance with the recommendations of the Declaration of Helsinki with written informed consent from all subjects. The protocol was approved by the Seattle Children’s Hospital and the University of Washington Institutional Review Boards.

## Author Contributions

MY conducted the tests and wrote the manuscript. MM evaluated the subjects. JR conceptualized the study, reviewed the data, and edited the manuscript.

## Conflict of Interest Statement

JR has served as a paid consultant to Cochlear Ltd., and Advanced Bionics Corporation, two manufacturers of cochlear implants. The remaining authors declare that the research was conducted in the absence of any commercial or financial relationships that could be construed as a potential conflict of interest.
